# Mechanisms and Αpplications of Ιnterleukins in Cancer Immunotherapy

**DOI:** 10.3390/ijms16011691

**Published:** 2015-01-13

**Authors:** Doxakis Anestakis, Savvas Petanidis, Spyridon Kalyvas, Christiane M. Nday, Olga Tsave, Efrosini Kioseoglou, Athanasios Salifoglou

**Affiliations:** 1 Laboratory of General Biology, Medical School, Aristotle University of Thessaloniki, Thessaloniki 54124, Greece; E-Mail: anestaki@auth.gr; 2 Laboratory of Forensic Medicine and Toxicology, Medical School, Aristotle University of Thessaloniki, Thessaloniki 54124, Greece; 3 Department of Chemical Engineering, Aristotle University of Thessaloniki, Thessaloniki 54124, Greece; E-Mails: spetanid@auth.gr (S.P.); christiane.nday@yahoo.com (C.M.N.); tsaveolga@gmail.com (O.T.); efi.kioseoglou@gmail.com (E.K.); 4 Department of Internal Medicine, General Hospital of Halkidiki, Poligiros 63100, Greece; E-Mail: roskal@otenet.gr

**Keywords:** interleukins, cancer immunotherapy, immunoediting, immunosurveilance, microRNA, cancer stem cells, tumor microenvironment (TME), inflammation, DNA methylation, epithelial-mesenchymal transition (EMT), autophagy

## Abstract

Over the past years, advances in cancer immunotherapy have resulted in innovative and novel approaches in molecular cancer diagnostics and cancer therapeutic procedures. However, due to tumor heterogeneity and inter-tumoral discrepancy in tumor immunity, the clinical benefits are quite restricted. The goal of this review is to evaluate the major cytokines-interleukins involved in cancer immunotherapy and project their basic biochemical and clinical applications. Emphasis will be given to new cytokines in pre-clinical development, and potential directions for future investigation using cytokines. Furthermore, current interleukin-based approaches and clinical trial data from combination cancer immunotherapies will also be discussed. It appears that continuously increasing comprehension of cytokine-induced effects, cancer stemness, immunoediting, immune-surveillance as well as understanding of molecular interactions emerging in the tumor microenvironment and involving microRNAs, autophagy, epithelial-mesenchymal transition (EMT), inflammation, and DNA methylation processes may hold much promise in improving anti-tumor immunity. To this end, the emerging in-depth knowledge supports further studies on optimal synergistic combinations and additional adjuvant therapies to realize the full potential of cytokines as immunotherapeutic agents.

## 1. Introduction

**Figure 1:**
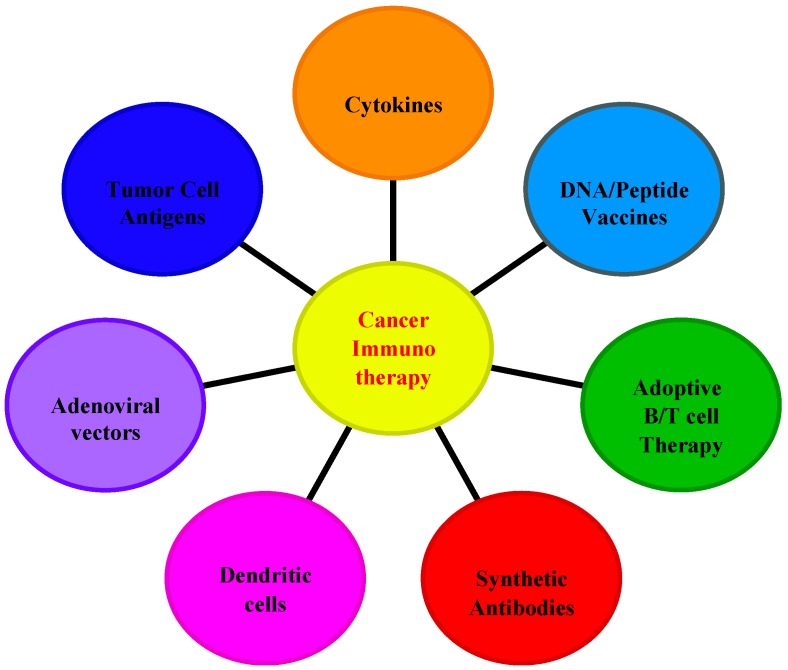
Current immunotherapeutic strategies used in cancer treatment.

Interleukins were first discovered in the 1970s as a subset of a broader group of cellular messenger molecules, the cytokines, which allow cells of the immune system to communicate with each other and generate a coordinated, specific, response to a target antigen [1,2,3]. Normally, interleukins are secreted by immune system cells in order to locate a targeted “hostile” cell and attach to it via specific receptors on the cell surface [4,5]. This attachment triggers a cascade of events within the target cell that ultimately alter the cell’s behavior. Furthermore, interleukins possess a variety of immunomodulatory functions that guide the immune system cell’s maturation, differentiation, migration and adhesion [6,7]. In tumorigenesis, these cytokines directly stimulate immune effector and stromal cells at the tumor site and enhance tumor cell recognition by cytotoxic effector cells. Recent studies have demonstrated that interleukins are involved in many tumor-driven molecular mechanisms and this has been translated into a number of cytokine-based approaches for cancer therapy (Figure 1). Over the last decade, a number of interleukins (IL), including IL-2, IL-7, IL-12, IL-18 and IL-21, have entered clinical trials for patients at the advanced cancer stage [8,9]. In addition, advances in cancer cell immunotherapy have relied on the use of cytokines to create an *in vitro* highly controlled environment for optimal development of anti-tumor T cells [10,11]. As tumors progress and evolve, they “try” to evade recognition by the immune system, essentially through creation of a “safe” tumor microenvironment [12]. In addition, tumors can secrete factors that suppress T cell responsiveness. This includes the expression of immune suppressive or anti-inflammatory cytokines, such as transforming growth factor-β (TGF-β) and IL-10, as well as enzymes such as arginase and indoleamine-2,3-dioxygenase (IDO) that catabolize amino acids critical for T cell effector functions [13,14]. Another trick that tumors use to escape the adaptive immune response is recruiting or converting inflammatory cells that suppress T cell responses. This includes regulatory T (Treg) cells, myeloid-derived suppressor cells (MDSC), and dendritic cells (DC) [15,16]. Likewise, interleukin family members present in the tumor microenvironment interact with various biomolecules, such as cancer stem cells, microRNA, epithelial-mesenchymal transition (EMT) markers and transcription factors. Consequently, involvement of interleukins in tumor-promoting mechanisms like DNA methylation, autophagy, immunoediting, immunosurveillance and inflammation-driven carcinogenesis stands as a well-defined platform for research into future effective and efficient cancer immunotherapy.

## 2. Interleukins

### 2.1. Function and Regulation

**Figure 2:**
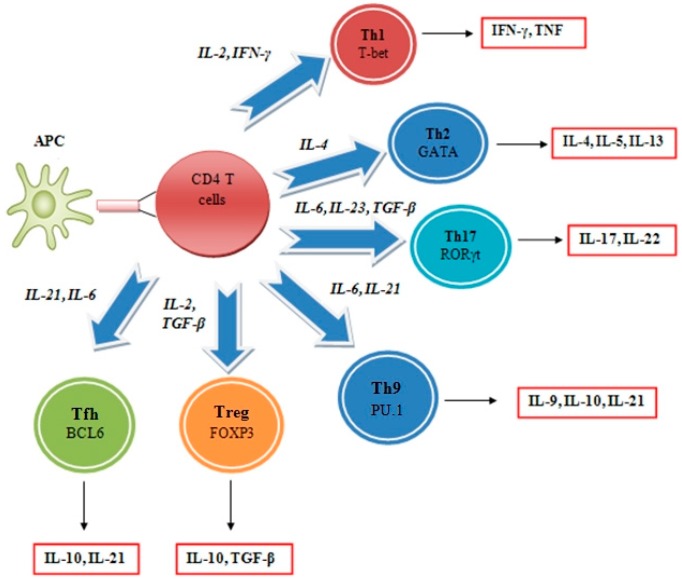
Following recognition of a specific antigen presented by an appropriately activated antigen-presenting cell (APC), CD4^+^ T cells can differentiate into different subsets determined by the cytokine milieu present when the cell encounters an antigen. These subsets are classified according to the dominant transcription factor in concert with cytokines that they express.

Cytokine interleukins belong to a family of immunomodulatory proteins that elicit a wide variety of responses in various tissues and organs [2,17]. These agents initiate an immune response by binding to high-affinity receptors and this response is dependent on the ligands involved, the specific receptors expressed on the cell surface and the particular signaling cascades activated. Nearly all interleukins modulate growth, differentiation and activation during an immune response [18,19]. Likewise, interleukins can exert both inflammatory and anti-inflammatory actions and can act as chemoattractants for T helper cells, sustaining an immune response [20]. Furthermore, many members of the interleukin family are intimately involved in the cellular defense against viral pathogens, and are very important mediators of the physiological response to infection, contributing significantly to the pathophysiology of a wide range of diseases [21]. As such, interleukins can function as potential therapeutic targets. The immune system is now known to be a key mechanism preventing the occurrence of cancer through processes involving the concept of immunosurveillance and immunoediting [22,23,24]. However, immunity fails in controlling tumor growth and metastasis, because of strong escape mechanisms developed by the tumor [25]. In recent years, several interleukin-based cancer immunotherapy strategies have been developed that target escape mechanisms of the tumor machinery [26,27]. This review provides an understanding of the mechanisms involved and identifies innovative therapeutic strategies. The majority of interleukins are secreted by the immune system cells cluster of differentiation 4^+^ (CD4^+^) T helper (Th) lymphocytes as well as through monocytes, macrophages, and endothelial cells (Figure 2).

These CD4^+^ T helper lymphocytes are important for the regulation of immune responses and characterized by their ability to modulate the function of CD8^+^ cytotoxic T lymphocytes, B cells, natural killer (NK) cells, macrophages, and dendritic cells [28]. CD4^+^ T cells are divided into distinct subgroups according to cytokine profile. In detail, CD4^+^ T cells can differentiate into Th1, Th2, Treg, Th17, TH22 and T-follicular effector cells [29,30,31]. Depending on the immune cell interactions, these T-cell subsets can endorse different types of immune-specific responses [32].

### 2.2. Interleukin Regulation of the Tumor Microenvironment

Recent advances in tumor immunogenetics have implicated the role of the tumor microenvironment in tumor progression and its association with interleukin-related immune response and immune regulation [33,34,35]. Recently, a tumor microenvironment macrophage inhibitory factor was found to promote accumulation of interleukin-17-producing tumor-infiltrating lymphocytes along with production of high levels of IL-6, IL-8, CD154^+^, granulocyte-colony stimulating factor (G-CSF) and chemokine (C–X–C motif) ligand 1 (CXCL1) [36]. Furthermore, it was shown that IL-12 with potent immunostimulatory activity and anti-angiogenic characteristics modulated hepatitis B virus x protein (HBx)-mediated intervention of hepatoma microenvironment, specifically pertaining to the intervention of neovessels and immune microenvironment. Treatment with IL-12 not only induced massive accumulation of immune cells, like CD8^+^ T leukocytes, macrophages, and dendritic cells in tumors *in situ*, but it also apparently reduced the number of angiogenic blood vessels within tumor tissues. These results showed that IL-12 can induce cell cycle arrest, apoptosis in human hepatocellular carcinoma (HCC) cells, and effectively shift the tumor microenvironment from pro-oncogenic to antitumor through recruitment of immune cells and inhibiting stromal cell growth [37]. Besides, IL-23, the Th17 cell survival factor, was also overexpressed in tumor tissues isolated from mice and human breast cancer patients, and tumor-secreted prostaglandin E2 (PGE2) induced IL-23 production in the tumor microenvironment, leading to Th17 cell expansion [38]. In addition, another interleukin, IL-18, primed “helper” NK cells to produce high levels of the immature dendritic cell (iDC)-attracting chemokines CCL3 and CCL4 upon exposure to tumor cells or the additional inflammatory signals IFN-α, IL-15, IL-12 or IL-2. These “helper” NK cells potently attract iDCs in a CCR5-dependent mechanism and induce high DC production of CXCR3 and CCR5 ligands (CXCL9, CXCL10 and CCL5), thereby facilitating the ensuing recruitment of type-1 effector CD8^+^ T (Teff) cells in the tumor microenvironment.

Another important cytokine located in the TME, IL-21, present in the bone marrow of patients with Waldenstrom macroglobulinemia (WM) significantly increased both IgM secretion and cellular proliferation of these cells with no effect on viability. IL-21 rapidly induces phosphorylation of signal transducer and activator of transcription 3 (STAT3) in WM cells. Treatment of the WM cell line MWCL-1 with a STAT3 inhibitor abolished the IL-21-mediated rise in cellular proliferation and IgM secretion. IL-21 also increased the expression of known STAT3 targets involved in B-cell differentiation, including BLIMP-1, XBP-1, IL-6 and IL-10. The data indicate that IL-21 in the bone marrow microenvironment significantly affects the biology of WM tumor cells through a STAT3-dependent mechanism [39]. Thus, antitumor strategies targeting specific interleukins in the tumor microenvironment could serve as new targets in the development of anticancer immunotherapy.

## 3. Interleukin Mechanisms Involved in Carcinogenesis

### 3.1. Crosstalk between Cancer Stem Cells and Cytokine Interleukins

An important characteristic role of interleukins is their ability to moderate cancer stem cell differentiation. IL-22 in cancer cells can promote activation of the transcription factor STAT3 and expression of the histone 3 lysine 79 (H3K79) methyltransferase DOT1L. The DOT1L complex induces the core stem cell genes *NANOG*, *SOX2* and *Pou5F1*, thereby resulting in increased cancer stemness and tumorigenic potential. Thus, IL-22(+) cells promote colon cancer stemness via regulation of stemness genes that negatively affect patient outcome [40]. Also, IL-6 is capable of generating CD44^+^ cells with stem-like properties through induction of the EMT in the T47D breast cancer cell line. The mammosphere cultures of epithelial-like breast cancer cells, T47D, MCF7, ZR-75-1 and MDA-MB-453 cells, consistently generate stem-like cancer cells solely as a result of the EGF and bFGF cytokines in the mammosphere media mediating EMT. This finding has demonstrated the link between the inflammatory cytokine IL-6 and BrCSCs and identified an important mechanism for the enrichment of BrCSCs in mammosphere cultures. Thus, IL-6 is capable of generating CD44^+^ cells with stem-like properties through induction of EMT in the epithelial-like T47D breast cancer cells. Hence, EMT appears to be a critical mechanism for the induction of cancer cells with stem-like properties [41]. On an equal footing, stromal interleukin IL-6 defines the tumorigenic capacity of cancer stem cells (CSC) sorted from primary human head and neck squamous cell carcinoma (HNSCC) and transplanted into mice. This points to a direct correlation between IL-6 levels in tumor-associated endothelial cells and the tumorigenicity of CSC. *In vitro*, endothelial cell-IL-6 enhanced orosphere formation, p-STAT3 activation, survival, and self-renewal of human CSC. Notably, a humanized anti-IL-6R antibody (tocilizumab) inhibited primary human CSC-mediated tumor initiation. Collectively, these data (a) demonstrate that endothelial cell-secreted IL-6 defines the tumorigenic potential of CSC; and (b) suggest that HNSCC patients might benefit from the therapeutic inhibition of IL-6/IL-6R signaling [42]. Another cytokine involved in cancer stem cell regulation is IL-1β, which can increase the sphere-forming capability of colon cancer cells in a serum-free medium. IL-1β-induced spheres exhibited up-regulation of stemness factor genes *Bmi1* and *Nestin* and increased drug resistance, all hallmarks of CSCs. Importantly, expression of EMT activator Zeb1 was increased in IL-1β-induced spheres, indicating that there might be a close association between EMT and IL-1β-induced CSC self-renewal. Indeed, IL-1β treatment led to EMT of colon cancer cells with loss of E-cadherin, up-regulation of Zeb1, and gain of the mesenchymal phenotype. Furthermore, shRNA-mediated knockdown of *Zeb1* in HCT-116 cells reversed IL-1β-induced EMT and stem cell formation [43]. Recently a study implicated IL-17 in promoting self-renewal of ovarian CD133^+^ cancer stem-like cells (CSLCs). IL-17-producing cells, CD4^+^ cells and CD68^+^ macrophages were detected in populations of CD133^+^CSLCs. Also, there was IL-17 receptor expression on CD133^+^CSLCs derived from the A2780 cell line and primary ovarian cancer tissues. Through recombinant human IL-17 stimulation and IL-17 transfection, the growth and sphere formation capacities of ovarian CD133^+^CSLCs were significantly enhanced in a dose-dependent manner. Moreover, ovarian CD133^+^CSLCs transfected with IL-17 showed greater tumorigenesis capacity in nude mice. These data suggest that IL-17 promoted self-renewal of ovarian CD133^+^CSLCs [44].

Overall, the aforementioned data suggest that certain interleukins, including IL-17, IL-1β and IL-6 are involved in mechanisms of stemness, which has been reported to be a promising pathway toward cancer immunotherapy. Therefore, significant promise is held in further studies deciphering the potential role that the rest of the interleukin family members might have in interwoven interactions with the aforementioned as well as other transcription factors. Sculpting the immune response may thus be a goal toward future cancer immunotherapies.

### 3.2. Molecular Interplay of MicroRNA with Interleukins

An important field of cancer immunotherapy involves regulation of microRNA expression by interleukins during cancer progression. MicroRNAs (miRNAs) are small non-coding RNAs, which can regulate gene expression post-transcriptionally [45,46]. The majority of miRNAs target about 80% of the protein-coding mRNAs and therefore can be considered master regulators of multiple cellular pathways, contributing to fine-tuning of the cell’s most important processes, including cellular growth, proliferation and differentiation. Deregulation of miRNAs plays a fundamental role in the onset, progression and dissemination of many cancers; therefore, impairment of miRNA biosynthesis is an important event in the tumorigenic cascade [47,48]. Specifically, miR-205 as a tumor suppressor was significantly lower in KB oral cancer cells than in human normal oral keratinocytes. Transfection of miR-205 into KB oral cancer cells strongly induced IL-24, a well-known cytokine acting as a tumor suppressor in a range of tumor tissues. In addition, miR-205 targeted the *IL-24* promoter directly to induce gene expression [49]. In another study, micro-RNA-205 induced the expression of tumor suppressor genes *IL-24* and *IL-32* at both the messenger RNA and protein levels. Induction of *in vitro* transcription and enrichment of markers for transcriptionally active promoters in the *IL-24* and *IL-32* genes was observed in response to miR-205 [50]. Likewise, certain miRNAs that regulate TGF-β induction of IL-11, also, mediate the bone metastatic process. For example, miR-204, miR-211 and miR-379 were shown to directly target *IL-11* by binding to its 3'-UTR. MiR-379 also inhibited Smad2/3/4-mediated transcriptional activity. Concurrently, miR-204, -211, and -379 reduced not only IL-11 secretion but *IL-11* mRNA levels as well. Furthermore, these results indicated that miR-204 and -211 bind to the *IL-11* 3'-UTR, an event in line with the predictions of several different bioinformatics algorithms projecting *IL-11* as a direct target for miR-204 and -211 [51]. In addition, IL-1β induces up-regulation of miR-425, which negatively regulates phosphatase and tensin homolog expression by targeting its 3'-UTR. An increase in miR-425 depends on IL-1β-induced NF-κB activation, which enhances miR-425 gene transcription upon IL-1β induction. Consequently, repression of phosphatase and tensin homolog by miR-425 promotes gastric cancer cell proliferation, which is required to protect cells from cisplatin-induced apoptosis. That NF-κB-dependent up-regulation of miR-425 represents a new pathway for the (a) repression of phosphatase and tensin homolog activation; and (b) promotion of cell survival following IL-1β induction [52]. Along the same lines, up-regulation of MMP-13 expression by IL-1β was correlated with down-regulation of miR-127-5p expression in human chondrocytes. The specific microRNA suppressed IL-1β-induced MMP-13 production as well as the activity of a reporter construct containing the 3'-UTR of human *MMP-13* mRNA. In addition, mutation of the miR-127-5p binding site in the 3'-UTR of *MMP-13* mRNA abolished miR-127-5p-mediated repression of reporter activity. Conversely, treatment with anti-miR-127-5p remarkably increased reporter activity and MMP-13 production. In contrast, IL-1β-induced activation of JNK, p38, NF-κB, and expression of MMP-1 and cyclooxygenase-2 were significantly inhibited by miR-127-5p [53].

Collectively, miRNA regulation of the expression of interleukins (IL-11, IL-1β, IL-24 and IL-32) emerges as a useful tool in probing modulatory mechanisms of cancer promotion/suppression and their (in)direct implication in immunotherapeutic approaches.

### 3.3. Association of Epithelial Mesenchymal Transition with Interleukin Expression

In recent years, therapeutic strategies in cancer immuno-therapeutics have focused on the Epithelial Mesenchymal Transition (EMT) phenotype of tumor cells. As a term, EMT refers to the process during which epithelial cells lose their polarized organization and cell to cell adhesion, undergo changes in cell shape and cytoskeletal organization, and acquire mesenchymal characteristics, such as increased cell migration and invasion [54]. It is now known that members of the interleukin family influence the EMT environment and shape the tumorigenic response. Interleukins such as IL-4 and IL-17A provide a Th2/Th17-polarized inflammatory milieu in favor of TGF-β1 to induce bronchial EMT. A synergic action was noted between TGF-β1, IL-4 and IL-17A, in terms of induction of EMT. Specifically, IL-4 and IL-17A synergized with TGF-β1 to (a) induce epithelial cells re-entering the cell cycle; and (b) promote epithelial to mesenchymal morphological transition [55]. In a similar manner, IL-8 and VEGF mediated epithelial-mesenchymal transition and invasiveness via p38/JNK-ATF-2 signaling in A549 lung cancer cells. These changes were accompanied by enhanced motility, invasion, anchorage-independent growth and anoikis-resistance. IL-8 along with VEGF were found to play a major role in the cancer cell metastatic potential [56]. Another EMT-related cytokine, interleukin-6 induced EMT through signal transducer and activator of transcription 3 in human cervical carcinoma. IL-6 receptor (IL-6R) and STAT3 were highly expressed in human cervical squamous cell carcinoma (CSCC) tissues and the expression of EMT markers was reversed in well-differentiated and poorly-differentiated human CSCC. Additional experiments showed that IL-6 exposure in cervical carcinoma cell lines induced IL-6R and STAT3 expression, promoted cell growth, and altered cell morphology. Treatment of cervical carcinoma cell lines with IL-6 resulted in down-regulation of E-cadherin and up-regulation of vimentin [57]. More to the point, Bcl-2, when co-cultured with head and neck tumor cells (CAL27), significantly enhanced EMT-related changes in tumor cells, predominantly through secretion of IL-6. Treatment with recombinant IL-6 or stable IL-6 overexpression in CAL27 cells or immortalized oral epithelial cells (IOE) significantly induced expression of the mesenchymal marker vimentin, while repressing E-cadherin expression via the JAK/STAT3/Snail signaling pathway [58]. Similarly, IL-32β expression was positively correlated with tumor stage, size, and number of lymph node metastases. In addition, MDA-MB-231 breast cancer cells, expressing IL-32β, exhibited increased migration and invasion capacities. These enhanced capacities were associated with an increased expression of the EMT markers vimentin and Slug [59].

Undoubtedly, therefore, involvement of certain interleukins including IL-6, IL-4, IL-17A and IL-32β in certain EMT pathways signifies their specific contribution to metastatic processes, thereby warranting further work into the development of diagnostic and immunotherapeutic tools in cancer.

### 3.4. Modulation of the Autophagic Machinery by Interleukins

A key feature of malignant cells is dysregulation of the autophagic process. In this phenomenon, interleukins play a dual role by inhibiting or promoting autophagy during tumorigenesis. In prostate cancer PCA cells, IL-6 expression resulted in the induction of autophagy, with the autophagy pathway required for IL-6-induced neuroendocrine differentiation and chemoresistance of prostate cancer cells. That implies that autophagy is involved in PCA progression and plays a cytoprotective role when stimulated by IL-6 [60]. Along the same lines, cell autophagy was enhanced through release of IL-2, whereas IL-10 attenuated the effect, and cell-to-cell contact strongly enhanced lymphocyte-mediated autophagy. Importantly, cell-mediated autophagy promoted resistance from treatment modalities designed to eradicate tumor cells [61]. Conversely, expression of mda-7/IL-24 in leukemia cells induced autophagy, which was triggered by up-regulation of Beclin-1. These results suggested that mda-7/IL-24 protein interacts with Beclin-1. Class III PI3K/Beclin-1 complex was shown to be involved in the mda-7/IL-24-induced autophagy. Moreover, autophagy inhibition by the PI3K inhibitor wortmannin resulted in a reduced Beclin-1 expression and autophagosome formation associated with significantly enhanced cell death [62]. In addition, IL-1β can induce autophagy trypsin activation and decrease cellular viability in pancreatic acinar cells. These effects depend on impaired autophagy via intracellular calcium changes. Treatment of pancreatic cells AR42J with IL-1β triggered autophagy and the autophagic flux was impaired. Moreover, IL-1β induced calcium release from the ER [63]. In contrast, administration of IL-2 inhibited autophagic flux in patients with melanoma and renal cell carcinoma. The autophagy inhibitor chloroquine synergistically enhanced IL-2 immunotherapeutic efficacy and inhibited tumor growth in a dose-dependent fashion. This combination increased long-term survival, decreased toxicity associated with vascular leakage, and enhanced immune cell proliferation and infiltration in the liver and spleen. Also, chloroquine increased autophagic vacuoles and LC3-II levels inhibited oxidative phosphorylation, ATP production, promoted apoptosis, and cytochrome c release from mitochondria [64]. These findings show that interleukin-induced cell-mediated autophagy promotes cancer cell survival and may represent an important target toward the development of novel therapies. They also reveal the potential of targeting autophagy as part of a combined immunotherapeutic regime for various tumors.

### 3.5. Correlation between DNA Methylation and Interleukin Expression

Recent studies in cancer immunology have implicated the association between interleukin expression and alterations in DNA methylation status of genes related to tumorigenesis. For instance, recent data reveal that DNA methylation is frequent in promoter regions of *IL-1b*, *IL-6* and *IL-8* in lung cancer. These cancer cells have significantly different DNA methylation and mRNA levels than normal human epithelial cells. Furthermore, (a) the high DNA methylation status of *IL* promoters in lung cancer cells or tissues was associated with low mRNA levels; and (b) an inverse correlation between DNA methylation of *IL-1β*, *IL-6* and *IL-8* gene promoters and their corresponding mRNA levels was observed. These results highlight the role of epigenetic modifications in the regulation of the expression of key cytokines involved in the inflammatory response during lung cancer progression [65]. Nevertheless, there is evidence that IL-6-induced inflammation promotes tumorigenesis in oral cancer cells by altering global *LINE-1* hypomethylation. In addition, concurrent hypermethylation of multiple tumor suppressor genes by IL-6 suggests that epigenetic gene silencing may be an important consequence of chronic inflammation in oral cancer. The findings reveal the molecular association between DNA methylation and IL-based inflammation in cancer progression [66]. Moreover, IL-6 also induces *CYP1B1* and *CYP2E1* gene expression in HCT116 and SW480 cells. Regulation of CYP2E1 expression occurs via a transcriptional mechanism involving STAT3. In CYP1B1 regulation, IL-6 down-regulates CYP1B1-targeting microRNA miR27b through a mechanism involving DNA methylation. In clinical samples, expression of CYP1B1 and CYP2E1 was radically increased in malignant tissue overexpressing IL-6 compared with matched adjacent normal tissue. Thus, colonic inflammation in the presence of IL-6 is associated with neoplastic tissue, which can alter metabolic competency of epithelial cells by manipulating CYP2E1 and CYP1B1 expression through transcriptional and epigenetic mechanisms. This can lead to increased activation of carcinogens and DNA damage, thus promoting colorectal carcinogenesis [67]. Another interleukin, IL-20, and its receptors are frequently dysregulated in NSCLC. *IL-20RB* mRNA was significantly elevated in NSCLC tumors and protein levels of the receptors IL-20RB and IL-22R1 were significantly increased in NSCLC patient tumors. IL-20 and its receptors were found to be epigenetically regulated through histone post-translational modifications and DNA CpG residue methylation. Besides, treatment with recombinant IL-20 resulted in decreased expression of the VEGF family members at the mRNA level [68]. Collectively, DNA methylation associated with interleukin regulation (IL-1b, IL-6, IL-8 and IL-20) during carcinogenesis formulates a well-defined binary interactive model in future research toward therapeutics.

## 4. Applications of Interleukins in Cancer Immunotherapy

### 4.1. Interleukins in Cancer Immunoediting and Immunosurveillance

**Figure 3:**
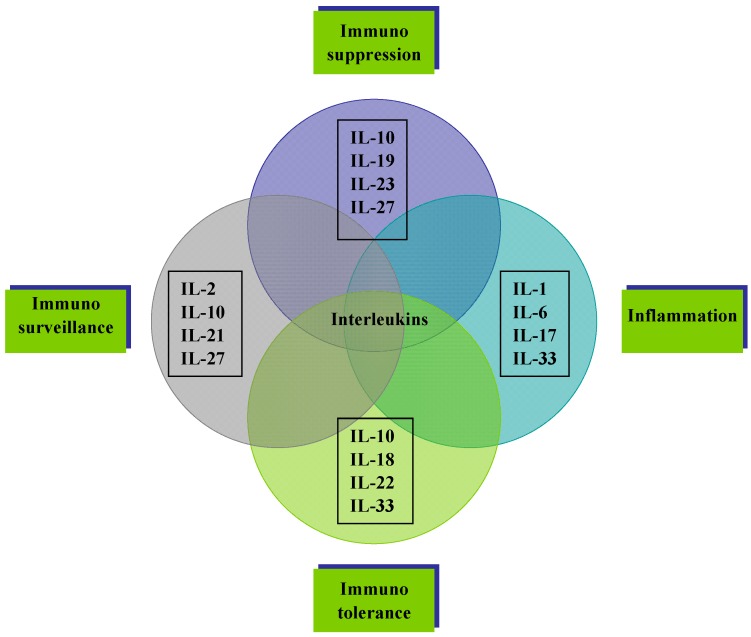
The major immune system-related interwoven roles of cytokine interleukins in the various stages of carcinogenesis.

Many members of the interleukin family participate in the involvement of immune system cells in tumor progression, by sculpting the immunogenic phenotype of tumors as they develop (Figure 3). Recognition that immunity plays a dual role in the tumor microenvironment (TME) interactions between tumors and the immune cells prompted a new dogma in cancer immunology, divided into three phases of cancer immunoediting: Elimination, equilibrium, and escape [69]. In the first phase, “elimination”, malignant cells are destroyed by immune system cells. The few tumor cells that manage to survive immune destruction then enter an “equilibrium” phase where molecular editing (mutations, gene rearrangement) takes place. During this stage, immune system cells and tumor cells live coexisting in the TME. The immune system, though not able to completely eliminate cancer, does not allow it to progress or metastasize further. In the third and final phase of this process, immunologically sculpted tumors begin to grow progressively and establish an immunosuppressive tumor microenvironment leading to uncontrolled carcinogenesis. In a recent study, innate immune cells exhibited cancer immunoediting activity in the absence of adaptive immunity. This activity required NK cells and IFN-γ, which mediated the induction of M1 macrophages. M1 macrophages could be elicited by administration of CD40^+^ agonists, thereby restoring editing activity in RAG2^−/−^ × γc^−/−^ mice. These results confirmed the fact that in the absence of adaptive immunity, NK cell production of IFN-γ induces M1 macrophages, which act as important effectors during cancer immunoediting [70]. Furthermore, cytokine interleukin 17D (IL-17D) was highly expressed in certain unedited tumors but not in edited mouse tumor cell lines. Moreover, forced expression of IL-17D in edited tumor cells induced rejection by stimulating MCP-1 production from tumor endothelial cells, leading to the recruitment of NK cells. These cells then promoted M1 macrophage development and adaptive immune responses. IL-17D expression was also lowered in certain high-grade and metastatic human tumors, suggesting that it can be targeted for tumor immune therapy [71].

Another cytokine, IL-21 coordinates colitis-associated tumorigenesis, leading to high IFN-γ and low IL-17A expression, which decreases tumor cell proliferation and increases tumor immunosurveillance. In experiments performed in tumors in IL-21-deficient mice, tumor cell proliferation (Ki-67) decreased, whereas cell apoptosis increased compared to wild-type mice. Increased IFN-γ expression in tumor-bearing IL-21-deficient mice caused increased tumor immunosurveillance, mediated by cytotoxic CD8^+^/CD103^+^ T cells targeting E-cadherin colonic tumor cells and therefore limited tumor growth [72]. In contrast, IL-4 aids cancer-initiating cells (CICs) responsible for tumor initiation, propagation, and resistance to chemotherapy, to escape from T cell-mediated immunosurveillance through membrane-bound IL-4 in colorectal cancer patients. CIC-associated IL-4 was found to be responsible for the production of high levels of immunomodulatory molecules, such as IL-4, and CIC-mediated inhibitory activity to anti-tumor T cell responses [73]. Taken together, these observations targeting interleukin family members responsible for immunoediting and immunosurveillance could increase knowledge targeting current cancer therapeutics.

### 4.2. Linking Interleukin Expression and Inflammation-Driven Carcinogenesis

Interleukin 17 family members participate in both acute and chronic inflammatory responses, which promote tumorigenesis and consist of cytokine members sharing amino acid sequence homology. Since IL-17A was discovered in early 1993, five other members of this family IL-17B, IL-17C, IL-17D, IL-17E and IL-17F have been recognized. The most important member of this family, IL-17A, is a pro-inflammatory cytokine playing a vital role in host defense against microbial infections and implicated in several inflammatory cascades including autoimmune diseases, metabolic disorders, and tumorigenesis [74]. IL-17A is responsible for the production of a variety of molecules including chemokines, matrix metalloproteinases and cytokines, which promote cascades of events that lead to inflammation, neutrophil recruitment, and host immune defense. Overexpression of IL-17A leads to severe inflammatory reactions and potential tissue damage. It is mainly produced by the T helper 17 (Th17) lineage as a regulatory cytokine contributing to the pathogenesis and maintenance of autoimmune and immune-inflammatory disorders. Aberrant expression of IL-17 is associated with several immuno-inflammatory disorders, such as multiple sclerosis (MS), rheumatoid arthritis (RA), inflammatory bowel disease (IBD), and psoriasis. Thus, development of therapeutics targeting suppression of IL-17 levels is a key approach to handling various inflammatory diseases. Over the past years, several anti-IL-17 and anti-IL-17 receptor antibodies have been under development for the treatment of autoimmune and chronic inflammatory diseases. For example, the anti-IL-17 antibody secukinumab and the anti-IL-17RA antibody brodalumab significantly improved clinicopathological symptoms of psoriasis. The molecular mechanism of these inhibitors targets binding of IL-17RA to ACT1. Binding to the IL-17R complex induces signaling via a distinct pathway that depends on the SEFIR-containing adapter protein ACT1 [75]. Both IL-17RA and IL-17RC subunits are required for the interaction with ACT1 and promote downstream signaling. Next, ACT1 mediates the recruitment of TRAF-6 (TNF receptor associated factor 6) and TRAF3, which are needed for activation of the nuclear factor-κB (NF-κB) pathway. IL-17A alone, however, is a weak NF-κB activator. What makes it, however, such a pathogenic cytokine is its ability to synergize with other cytokines like TNF-α to promote and prolong pro-inflammatory responses. IL-17, produced locally in the tumor microenvironment, plays important roles in both angiogenesis and tumor immunity. Inhibition of IL-17A at tumor sites by intratumoral injection of an adenovirus vector expressing siRNA against the mouse *IL-17A* gene (Ad-si-IL-17) significantly inhibited tumor growth. It was found that inhibition of IL-17 at tumor sites significantly suppressed CD31^+^, MMP9 and VEGF expression in tumor tissue. Furthermore, the cytotoxic activity of tumor infiltrating CD8^+^ lymphocytes in mice treated with Ad-si-IL17A was significantly higher than in control mice [76]. Further experimental evidence supports the idea that IL-17 promotes tumor growth. Specifically, ablation of IL-17 significantly reduced tumor development in mice bearing a heterozygote mutation in the adenomatous polyposis coli (APC) gene (*Apc^Min/+^* mice). There was also a decrease in inflammatory cytokines and proinflammatory mediators and reduction in lymphocytic infiltration, suggesting that IL-17 promotes spontaneous intestinal tumorigenesis [77]. Consistent with a positive role of IL-17 in promoting tumor development, tumor tissues have a higher frequency of IL-17^+^ T cells compared with untransformed bowel tissues [78]. The role of IL-17 in promoting tumor growth provides additional support for the already well-established connection between inflammation and tumorigenesis [79]. However, this study did not identify whether Ad-si-IL-17 induced IL-17 ablation only in T effector cells or its effects were also extended to Treg cells, a population also capable of producing IL-17 and intimately linked to the development of inflammation and cancer in the bowel. The above advances have contributed to understanding of the cellular and molecular pathways involved in cancer-related inflammation and stand as the foundation for further investigations linking interleukin involvement to inflammation-driven carcinogenesis.

### 4.3. Strategies for IL-Based Cancer Immunotherapy

Currently, certain interleukins are being used for targeting tumor cells with quite promising results. Interleukin-24 (IL-24) has been suggested as such an effective anticancer agent. In a latest study, the effects of IL-24 delivered by mesenchymal stem cells (MSCs) as a therapeutic approach for lung cancer were evaluated. Human umbilical cord-derived MSCs (UC-MSCs) were used to efficiently deliver secretable IL-24. IL-24-transduced UC-MSCs (IL-24-MSCs) inhibited growth of A549 lung cancer cells by induction of apoptosis and cell cycle arrest. The IL-24 proteins secreted by IL-24-MSCs were involved in regulating the ERK-1/2, AKT and JNK signaling pathways. Additionally, MSC-mediated IL-24 expression led to an increase in the cleavage of caspases-3/8/9 and PARP, the Bax/Bcl-2 ratio, as well as p21 expression in A549 cells. Furthermore, injection of IL-24-MSCs significantly suppressed xenograft tumor growth in mice [80]. In addition, recombinant MDA-7/IL-24 was shown to be selective against cancer cells from colorectal cancer (CRC) patients. Thus, MDA-7/IL-24 caused cellular apoptosis via a p53-independent manner, accompanied by cell cycle arrest in G0/G1 through down-regulation of cyclin D1 levels, and apoptosis induction through up-regulation of cell surface-bound Fas/CD95^+^ [81].

Besides, the cytokine member IL-22, which is mainly secreted by the T cells subsets, Th17 and Th22 subsets and innate lymphoid cells (ILCs), has also been used in cancer immunotherapy. IL-22 signals through its receptors IL-22R1 and IL-21R2 to activate members of the STAT family (STAT 1/3/5), PI3K, Akt, MAPK and mTOR signaling pathways. Immunotherapeutic strategies to inhibit the IL-22-IL-22R1 complex include molecules that block chemokines, which attract IL-22-producing cells, such as the inflammatory chemokine (C–C motif) ligand 20 (CCL20) or antibodies that neutralize mediators supporting IL-22 production of these cells, like IL-23 or TNF. In this sense, TNF and IL-23 are key inducers of IL-22 production and TNF augments the effects of IL-22. TNF blockers are already in clinical use (infliximab, etanercept, certolizumab and golimumab), attempting to prevent the signaling action of TNF, thereby down-regulating the expression levels of IL-22 in many inflammatory diseases, such as Crohn’s disease, rheumatoid arthritis, ulcerative colitis, psoriatic arthritis, ankylosing spondylitis and juvenile idiopathic arthritis.

In a recent publication, the recombinant Newcastle disease virus (rNDV) was used to enhance the cancer therapeutic potential. NDV was engineered to contain both interleukin-2 (IL-2) and tumor necrosis factor-related apoptosis inducing ligand (TRAIL). The findings showed that rNDV-IL-2-TRAIL significantly enhanced the inherent anti-neoplastic activity of rNDV by inducing apoptosis and increasing apoptosis-related gene mRNA expression. It also (a) promoted proliferation of the CD4^+^ and CD8^+^ in both hepatocellular carcinoma and melanoma-bearing mice; and (b) elicited expression of TNF-α and IFN-γ antitumor cytokines [82]. The collective data reflect apt examples of interleukin-based approaches in cancer immunotherapy and related diseases.

### 4.4. Combination Cancer Immunotherapy

**Figure 4:**
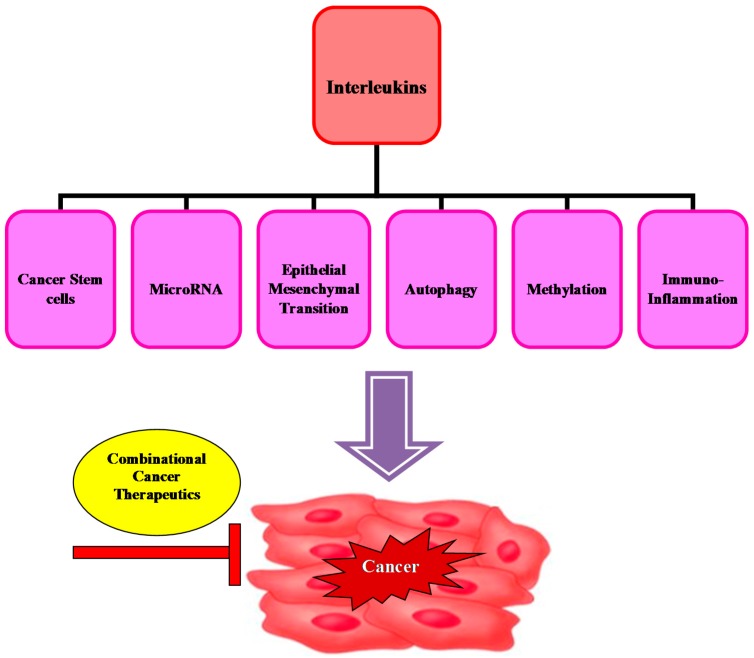
Combinational cancer therapeutics target interleukin interplay with key molecular mechanisms involved in tumorigenesis.

Although current cancer immunotherapy approaches have a positive clinical outcome, the antitumor efficacies of these therapies are still limited, due to the high degree of cancer clonal heterogeneity and intratumor genetic heterogeneity. For this reason, the use of combination cancer immunotherapeutic has been proposed in an attempt to improve the anticancer efficacy of traditional cancer therapeutics (Figure 4).

Clinical data indicate that autologous dendritic cell vaccines combined with IL-2 decreased TGF-β and CD4^+^CD25^+^ T cell levels and increased IL-12p70 levels in renal cell carcinoma or breast cancer patients. These combinatorial alterations of immunological parameters, indicating antigen-specific immune induction along with reduction of inhibitory immunity, were correlated with clinical responses in DC vaccine-treated patients [83]. In addition, the efficacy of combination therapy using adeno-associated virus-mediated (AAV) co-expression of apoptin and interleukin-24 on hepatocellular carcinoma is also promising. The findings reveal that AAV-mediated co-expression of IL-24 and apoptin significantly suppresses growth and induces apoptosis in HepG2 cells *in vitro*. Furthermore, AAV-mediated combined treatment of IL-24 and apoptin significantly reduces tumor growth and induces apoptosis of cancer cells in nude xenograft mice [84]. Furthermore, combination therapy of IL-15 and mTOR inhibitor everolimus inhibits breast cancer metastasis. This approach has been shown to increase the proportion of CD4^+^ T and NK cells but had no effect on CD8^+^ T cells. Both IL-15 and everolimus decreased expression of Ki-67 and increased apoptotic rates. Although both molecules are effective, no synergistic effect was observed with a combined treatment of everolimus and IL-15 gene therapy [85]. In addition, combination therapy of intratumoral IL-12, human tyrosinase (hTyr) DNA vaccination and metronomic cyclophosphamide (CPX) has shown increased antitumor effects in a B16-F10 mouse melanoma model. All treatment groups showed increased survival, higher cure rates than control groups, and significantly lower percentages of regulatory T cells [86]. Along these lines, heat shock protein vaccination and directed IL-2 therapy amplify tumor immunity rapidly following bone marrow transplantation in mice. This combination therapy resulted in a marked prolongation of survival, which correlated with an increase in effector CD8^+^ T-cell numbers and elicited large increases in both donor CD8^+^ T and NK cells, but not CD4^+^ T lymphocytes [87]. The above successful trials on combinatorial therapies such as cell vaccines with IL-2, AAV co-expression of apoptin and IL-24, IL-15 and everolimus, combination therapy of intratumoral IL-12, human tyrosinase (hTyr) DNA vaccination and metronomic cyclophosphamide (CPX), heat shock protein vaccination and directed IL-2 therapy, warrant further work into the development of target-specific cancer immunotherapy treatment(s).

## 5. Conclusions

Antitumor strategies targeting specific interleukins in the tumor microenvironment could serve as new targets in the development of anticancer immunotherapy. The aforementioned data suggest that certain interleukins, including IL-17, IL-1β and IL-6 are involved in key mechanisms of tumorigenesis, reported to be a promising pathway toward cancer immunotherapy. Therefore, significant merit is held toward further studies attempting to decipher the potential role that the rest of the interleukin family members might have in interwoven interactions with the aforementioned as well as other transcription factors. Concurrently, miRNA regulation of the expression of interleukins (IL-11, IL-1β, IL-24 and IL-32) emerges as a useful tool in probing modulatory mechanisms of cancer promotion/suppression and their (in)direct implication in immunotherapeutic approaches. In addition, the involvement of certain interleukins including IL-6, IL-4, IL-17A and IL-32β in some EMT pathways signifies their specific contribution in metastastic processes, thereby warranting further work into the development of diagnostic and immunotherapeutic tools in cancer. Interleukin-induced cell-mediated autophagy promotes cancer cell survival and may represent an important target for the development of novel therapies revealing the potential of targeting autophagy as part of a combined immunotherapeutic regime for various tumors. Also, interleukin family members responsible for immunoediting and immunosurveillance could increase the outcome in current cancer therapeutics. Numerous advances have contributed to the understanding of the cellular and molecular pathways involved in cancer-related inflammation and stand as the groundwork toward further investigations linking interleukin involvement to inflammation-driven carcinogenesis. Sculpting the immune response may thus be a goal toward future cancer immunotherapies. The collective data mentioned in the current review reflect apt examples of interleukin-based approaches in cancer immunotherapy and related diseases. To this end, better understanding of the molecular signaling pathways used by interleukin interjection in TME, stem cells, microRNA, epithelial mesenchymal transition, DNA methylation, autophagy, immunoediting and immunosurveillance, inflammation-driven carcinogenesis and immune cells, stands as a well-defined platform for research into future effective and efficient cancer immunotherapy. Defined into such a well-formulated framework, interleukin-based approaches in cancer immunotherapy deserve due attention and warrant further investigation.
